# The continuing HIV vaccine saga: naked emperors alongside fairy godmothers

**DOI:** 10.1186/1476-9433-4-6

**Published:** 2005-05-06

**Authors:** Kendall A Smith

**Affiliations:** 1 The Division of Immunology, Department of Medicine, Weill Medical College, Cornell University, New York, NY USA

## Abstract

The latest developments in the HIV vaccine field were aired at a Keystone Symposium recently. This Commentary summarizes some of the highlights from this meeting, and focuses on some of the developments that appeared particularly promising, as well as those that do not. Unfortunately, the "saga" continues.

## Introduction

Investigators working in the HIV vaccine field congregated at a Keystone Symposia meeting that was held in Banff, Canada April 9–15, 2005. David Montefiori, and Kent Weinhold, both from Duke University, and Carolyn Williamson, from the University of Cape Town chaired the meeting, which was held concomitantly with a meeting on HIV Pathogenesis, chaired by Michael Lederman from Case Western University, Richard Koup from NIAID/NIH, and Michael Malim, from King's College London.

The meetings were noteworthy for new insights into the structure-activity relationships of the viral envelope, additional characterizations of the functional aspects of both CD4+ and CD8+ T cells in successful immune responses to HIV and other viruses and vaccines, as well as functional defects of these same cells from subjects with persistent viremia and progressive disease.

Against this scientific backdrop, was a thrilling presentation by Judy Lieberman of Harvard Medical School, who appears to have solved the problem of delivery of small-interfering RNA (siRNA) to specific target cells *in vivo*.

However, the various academic groups and companies who are competing to develop the first effective prophylactic HIV vaccines created the drama of the meeting, which was played out in several presentations and posters over the course of the 6-day meeting. Accordingly, this commentary will focus on these reports and developments in the hopes of providing a flavor of the proceedings to those who were unable to attend.

## The envelope

Bing Chen from Children's Hospital Laboratory of Molecular Medicine of Harvard Medical School presented data from a collaborative effort by his group and by Stephen Harrison's group at Harvard Medical School and by John Skehel from the National Institute of Medical Research The Ridgeway, Mill Hill, London. They have also recently published their findings [1,2]. As these investigators pointed out, structures of fragments of gp120 and gp41 from the envelope glycoprotein have been known for some time, in conformations corresponding to their states after attachment to CD4 molecules and after membrane fusion. By comparison, these investigators determined the crystal structure, at 4.0 Å resolution, of a fully glycosylated and unliganded SIV gp120 core protein, in a conformation representing its prefusion state, prior to interaction with CD4. Comparison of the new structure and the HIV gp120 core in the CD4-bound conformation revealed a striking structural rearrangement in parts of the protein, resulting in distinct antigenic surfaces. Their model predicts that upon binding to CD4, parts of gp120 will shift around the CD4-binding cavity with very large excursions, e.g. the tip of the V1-V2 stem moves over 40 Å. Their model predicts that until co-receptor binds, the V1-V2 stem is not docked against the β20-β21 ribbon from the outer domain, and thus the bridging sheet, which binds to the co-receptor is not properly formed.

All of these structure-activity aspects of the envelope were graphically depicted in molecular movies, which allowed the uninitiated to appreciate the huge distances that parts of the molecules travel after binding CD4, to form the co-receptor binding site. Hopefully, these new insights into the dynamic structure and activity of the envelope will provide for new approaches so as to craft molecular immunogens that will promote the generation of neutralizing antibodies.

## T Cell Exhaustion

There is a consensus among almost all investigators now, that persistent viral infection leads to a qualitative defect in both CD4+ and CD8+ T cells, which is manifest by an incapacity to produce cytokines, especially IL2, when activated *in vitro *by viral peptides. By comparison, cells from the same individual can respond fully and appropriately to other antigens to which the individual is immune, e.g. antigens from cytomegalovirus (CMV) and Epstein-Barr virus (EBV). The consequence of the inability to produce IL2 is a poor proliferative response and an inability to differentiate into an "effector" capacity, whether monitored by cytokine/chemokine production or by cytolytic capacity.

Michael Betts from the Vaccine Research Center at NIH monitored cytokine production by 11-parameter flow cytometry to examine five antigen-specific CD8+ T cell functions simultaneously (degranulation-CD107a; cytokine/chemokine expression-INF-γ, TNF-α, IL2, MIP1β) in 9 HIV-infected Long-Term Nonprogressors (LTNP) and 79 Progressors. The LTNP maintained a polyfunctional CD8+ T cell response, expressing most of the gene products assayed, while the progressors were markedly deficient. As well, the polyfunctional response was found to be a normal component of effector CD8+ T cell responses to CMV, EBV, and influenza virus in normal individuals. Very noteworthy was the finding that the polyfunctional responses were not confined to a particular surface phenotype, whether thought to reflect a "central memory" phenotype or not. These findings may well augur the end of phenotypic analysis as a surrogate for lymphocyte function.

Giuseppe Pantaleo seconded these findings, and emphasized that the lack of control of viral replication is not due to the lack of HIV-specific CD4+ or CD8+ T cells, as originally proposed. Instead, Pantaleo's data mirrored Betts' data, and he stressed that the capacity to produce IL2 correlates best with a good immune response, whether to HIV or to CMV or EBV, by either HIV-infected or uninfected individuals.

Accordingly, the $64 question still unanswered is why there seems to be such a specific defect in HIV-specific T cells and why it is manifest by the incapacity to express the IL2 genes. However, all of these data point clearly to where research must focus in the future, for not until this defect is understood will it be approachable therapeutically. It remains to be determined whether IL2 administration can circumvent the defect in IL2 production by HIV-specific CD4+ and CD8+ T cells.

In this regard, Mark Connors from NIAID/NIH reported on further data from his group indicating that in addition to a defect in IL2 production, CD8+ T cells from Progressors cannot respond to IL2 by proliferating *in vitro *in response to stimulation by HIV antigens. He emphasized that 10% to 40% of circulating CD8+ T cells are HIV-reactive when individuals are viremic, which is a huge proportion of the total CD8+ T cell mass. Using HIV-peptide tetramers to identify HIV-specific cells, he could show that the CD8+ T cells from both LTNP and Progressors are CD25-, and gene expression in these cells as monitored by DNA arrays are identical when studied de novo, before activation. However, when antigen activated, even the addition of exogenous IL2 does not cause CD8+ T cells from Progressors to divide. He suggested that his data point to a defect in IL2-responsiveness, which could be attributable to changes in the IL2 receptor chains, the IL2R signaling pathways, or in the expression of IL2-regulated genes, in particular the IL2-reguated genes that promote cell cycle progression. He further indicated that such qualitative defects in the virus-specific CD8+ T cell response remains the most likely determinant of the loss of immunological restriction of virus replication. As such, his data indicate that a further understanding of these qualitative features may provide important information on how these changes might be avoided in vaccinees or reversed in infected individuals.

## RNAi

Judy Lieberman presented breathtaking data, which indicated that it may be possible to use the siRNA technology to create effective microbicides, and as well to target only HIV-infected cells systemically *in vivo*, by formulating magic bullets to seek out and suppress infected cells. If her data hold up and can be repeated and substantiated, it is a quantum leap forward, not only for the prevention and therapy of HIV infection, but for almost all aspects of biomedical research and medicine.

As explained by Lieberman, RNAi is an ancient, evolutionarily conserved host defense against viruses and transposable elements, which uses small double-stranded RNAs, called small interfering RNAs (siRNA), to silence gene expression with high specificity by targeted degradation of homologous mRNAs [3]. Lieberman's group used a mouse model of Herpes Simplex Virus-2 (HSV-2) vaginal infection to develop an siRNA-based microbicide. When mixed with lipids, siRNAs are efficiently and uniformly taken up by mucosal and submucosal cells and effectively silence gene expression in the mouse vagina. siRNAs that individually silence a variety of HSV-2 genes and inhibit HSV-2 replication in vitro by 4-8-fold were identified. Vaginal installation of siRNAs targeting HSV-2 was well tolerated and protected mice from lethal HSV-2 infection.

In other experiments, Lieberman targeted HIV-infected cells with RNAi by using a chimeric molecule engineered to contain the antigen-binding region of an antibody reactive with gp120, and protamine, a positively charged molecule. When mixed with siRNA targeting HIV genes in aqueous solution, the negatively charged siRNA forms a stable complex with the antibody-protamine chimera. Subsequently she showed that this molecular complex would selectively bind to target cells that expressed gp120. Accordingly, the antibody is used to identify the correct cells and the protamine delivers the siRNA. The data presented by Lieberman suggest that it may now be possible to silence your gene of choice, thereby changing gene therapy from a discipline focused on expressing missing genes, to silencing unwanted gene expression! A paper is in press that describes these remarkable findings [4].

## The HIV vaccine pipeline (The Emperors and the Fairy Godmothers)

Standing in stark contrast to these promising developments on the basic science scene, were disappointing, perplexing, but then promising developments on the more practical aspects of developing effective vaccines.

Andrew McMichael from Oxford supported by the International AIDS Vaccine Initiative (IAVI) was perhaps one of the first entrants into to the race to develop an effective vaccine. Most everyone now knows that Oxford/IAVI discontinued their large phase I/II trial in Kenya last year. McMichael reviewed the reasons for this decision for the participants of the Keystone conference. The Oxford group chose a combination of a DNA prime immunization, followed by a booster immunization with Modified Vaccinia Ankara (MVA). At the time that this approach was planned, now several years ago, the prevailing opinion was that it is important to select "immunodominant" epitopes to include in the vaccines. Thus, both the DNA priming vaccine and the MVA boost vaccine only contained *gag *p24, p17, and a "string" of selected CTL epitopes from other regions of the HIV genome.

McMichael reported that the responses in the Kenyon trial were disappointing, with only ~20% of vaccine recipients giving a positive immune response to the immunization in a highly stringent validated IFN-γ ELISPOT assay. He went on to say that perhaps a better assay to detect immune reactivity to the vaccine antigens could be obtained by culturing the PBMCs from vaccinated people *in vitro *with HIV peptides and IL2 for 7–14 days before testing for IFN-γ producing cells by ELISPOT. He suggested that perhaps this "reactivated response" would be more representative of long-term memory. This may be true, but this type of "reactivated response" precludes the capacity to quantify the frequencies of HIV antigen-reactive T cells from a subject, because after 1–2 weeks of selective expansion of antigen-reactive cells in IL2-dependent growth, and the demise of antigen nonreactive cells, any attempt at quantification is fruitless.

Searching for possible reasons as to why this immunization strategy failed, the most probable are the lack of breadth of antigens included in the vectors, and the fact that both vectors are replication incompetent. For a discussion of replication competent vs. incompetent vaccines, see [5].

Perhaps the next entrant into the HIV vaccine race was the Merck Corporation, which also adopted a DNA priming concept, followed by a boost with replication incompetent Adenovirus serotype 5 (Ad5). Merck elected to include only genes encoding the *gag *polyprotein in their vectors. John Shiver summarized their findings when immunizing normal volunteers. Whether assayed by ELISPOT or by flow cytometry monitoring the accumulation of intracellular IFN-γ, the immune responses were almost undetectable, with frequencies of only 0.1%–0.2% by flow cytometry (1000–2000 spot-forming cells/million PBMCs by ELISPOT assay). Because these normal volunteers cannot be challenged with HIV to test their antiviral immunity, it remains unknown whether these immune responses will reflect protective immunity or not. However, at best these immune responses are just at the Lower Limit of Detection (LLD) by the flow cytometry assay. As well, it is known the majority of the people in the US are already immune to the Ad5 serotype, and to detect even marginal immune responses to the immunogen, a huge dose had to be given, 100 billion virus particles. It is not clear why Merck is pursuing this line of investigation, given these marginal results. Evidently there is no one at Merck willing to stand up and say that the Emperor has no clothes [6].

The last entrant into the HIV vaccine race that reported on data in humans was the Vaccine Research Center (VRC) of the NIAID/NIH, which is directed by Gary Nabel. The VRC has adopted an approach very similar to Merck, with a DNA prime followed by an Ad5 boost. However, in contrast to Merck, the VRC has placed more HIV genes into their vaccines. They are using a separate DNA plasmid for the *env *genes from clades A, B, and C. Then a separate plasmid contains the genes encoding the clade B *gag*, *pol*, and *nef *genes. This immunization is followed by a combination of 4 Ad5 vectors encoding clade B *gag *and *pol*, and *env *from clades A, B, and C.

Barney Graham reported on the findings available to date. Remarkably, despite the addition of more HIV genes in the vectors, the immune responses were still barely detectable by flow cytometry, on the order of 0.1% to 0.2% positive cells by intracellular cytokine staining. Again, these data were presented as if one were dealing with *strong *immune responses, not the marginal immune responses that they actually represent. Accordingly, there was no mention of discontinuing this approach.

Given these marginal immune responses elicited in normal individuals, one wonders whether a "Gatekeeper" should be used, such as prophylactic trials in monkeys as was suggested by some at the meeting, or by obtaining an antiviral endpoint in HIV-infected humans, prior to undertaking large scale prophylactic trials in developing countries [5,7].

Fortunately there are some Fairy Godmothers waiting in the wings, anxious to wave their magic wands to take over after the Emperors retreat to hide their nakedness. Harriet Robinson's group from Emory University submitted 5 abstracts that resulted in as many posters. Of these reports, one of the most interesting was a study where they mapped and characterized the T cell responses associated with long-term control of a virulent SHIV-9.6P challenge in 22 macaques vaccinated with a Gag-Pol-Env DNA/MVA vaccine. Thus, they have employed an approach similar to the Oxford group, but they have included the 3 most important/prevalent genes in their vaccine. In addition, the MVA that they use is different from the Oxford MVA, in that it releases virus-like particles (VLP) [8]. Therefore, it expresses more of a viral replicative cycle, so that it should be more immunogenic. They tested for the frequency, breadth, and stability of anti-viral CD4+ and CD8+ T cells and for the protection of T cell function as evidenced by IFN-γ and IL2 co-production during a 200-week period of viral control (3.9 years). Even 2.5 years post challenge, IFN-γ-producing T cells were still detectable in frequencies up to 1.0% of total CD8+ or CD4+ T cells, which is 10-fold greater than the frequencies that the Oxford, Merck and VRC groups are reporting in humans. Moreover, these T cell responses were remarkably stable when tested a year later and maintained good function, as evidenced by the co-production of IFN-γ and IL2.

This group also presented data in humans on a phase I multi-center, randomized, placebo-controlled, double blind trial conducted under the auspices of the HVTN. A clade B DNA HIV vaccine developed for use as a priming vector for a DNA/MVA combination vaccine in humans was tested in 30 HIV-uninfected normal volunteers. This 9.5 kb DNA uses a CMV promoter and a bovine growth hormone polyadenylation sequence to express a single transcript that undergoes Rev-dependent subgenomic splicing and frame-shifting to express Gag, Pr, RT, Env, Tat, Rev and Vpu. In this phase I safety trial, doses of 0.3 mg and 3.0 mg were well tolerated. ELISPOT assays performed on frozen cells were negative after 2 immunizations. However, flow cytometry assays performed on fresh cells did detect some responses, although at low frequencies, 0.01–0.1%. Additional data from this group will be awaited with interest.

Perhaps the maverick of the group is Marjorie Robert-Guroff from the NCI. She has been developing ***replication-competent ***Adenoviruses as vectors for HIV vaccines [9]. In contrast to the Merck and VRC/NIH groups, she has focused her attention on two different serotypes, #s 4 and 7. The U.S. Army had used these two serotypes as vaccines for new recruits between 1972 and 1996, and as many as 10 million young men received these vaccines, apparently safely. The immunizations were discontinued in 1996 because the company manufacturing them ceased production. However, a large majority of young adults in the U.S have not been exposed to these serotypes, so that they are at risk of developing severe upper respiratory infections. Because of this, and because of the Iraqi war, the government is now planning to reintroduce these vaccines. Since most individuals are not already immune to these serotypes, they make for prime candidates for use as vectors for vaccines against other microbes, such as HIV. Robert-Guroff is planning initial clinical trials with Ad4-based HIV recombinants.

A comprehensive analysis of the replication-competent Ad vaccine approach in the HIV-chimpanzee and SIV-macaque models demonstrated that it elicits potent humoral, cellular, and mucosal immunity yielding strong, durable protective efficacy against pathogenic SIV. Comparing replication-competent with replication-incompetent Ad vectors, she demonstrated that 10-100-fold lower doses of replication-competent Ad vectors elicited significantly more, and longer-lasting IFN-γ-secreting cells in response to env peptides compared with replication-incompetent Ad vectors, and primed stronger T cell proliferative responses. As well, in a combined Ad-HIV recombinant priming/protein boosting approach using Chiron's oligomeric HIV gp140ΔV2 vaccine, enhanced humoral immunity was elicited with the replicating Ad recombinants, with higher anti-envelope binding and neutralizing antibody titers, and greater capacity to neutralize a broad spectrum of heterologous HIV R5-tropic primary isolates. Replicating Ad-recombinants also elicited antibodies that mediated significantly greater levels of antibody-dependent cellular cytotoxicity (ADCC), and she showed data that the ADCC activity correlated with antiviral protection after SIV challenge [10].

Robert-Guroff concluded by stating that their results should support continued development of the replicating Ad-HIV recombinant vaccine approach, and suggested that greater attention should be focused on replicating vectors in general. As well, she pointed out that replicating vectors have a strong adjuvant effect and elicit persistent immunity, as evidenced by almost all of the live attenuated replication competent viral vaccines that have already been licensed. Finally, she stressed that *"the use of replication-competent vectors, alone or in combination with other replicating, or non-replicating vectors, coupled with well-designed envelope boosts, may provide the broad, potent immunity and rapid responsiveness necessary for an effective prophylactic HIV vaccine."*

Perhaps the Emperors should take note when designing their next suit of clothes!
